# Neuron to Astrocyte Communication via Cannabinoid Receptors Is Necessary for Sustained Epileptiform Activity in Rat Hippocampus

**DOI:** 10.1371/journal.pone.0037320

**Published:** 2012-05-15

**Authors:** Guyllaume Coiret, Jeanne Ster, Benjamin Grewe, Fabrice Wendling, Fritjof Helmchen, Urs Gerber, Pascal Benquet

**Affiliations:** 1 Brain Research Institute, University of Zurich, Zurich, Switzerland; 2 INSERM U1099, Laboratoire Traitement du Signal et de L'Image, University of Rennes 1, Rennes, France; Institut National de la Santé et de la Recherche Médicale, France

## Abstract

Astrocytes are integral functional components of synapses, regulating transmission and plasticity. They have also been implicated in the pathogenesis of epilepsy, although their precise roles have not been comprehensively characterized. Astrocytes integrate activity from neighboring synapses by responding to neuronally released neurotransmitters such as glutamate and ATP. Strong activation of astrocytes mediated by these neurotransmitters can promote seizure-like activity by initiating a positive feedback loop that induces excessive neuronal discharge. Recent work has demonstrated that astrocytes express cannabinoid 1 (CB1) receptors, which are sensitive to endocannabinoids released by nearby pyramidal cells. In this study, we tested whether this mechanism also contributes to epileptiform activity. In a model of 4-aminopyridine induced epileptic-like activity in hippocampal slice cultures, we show that pharmacological blockade of astrocyte CB1 receptors did not modify the initiation, but significantly reduced the maintenance of epileptiform discharge. When communication in astrocytic networks was disrupted by chelating astrocytic calcium, this CB1 receptor-mediated modulation of epileptiform activity was no longer observed. Thus, endocannabinoid signaling from neurons to astrocytes represents an additional significant factor in the maintenance of epileptiform activity in the hippocampus.

## Introduction

Synaptic transmission depends on complex interactions between presynaptic terminals, postsynaptic targets and associated astrocytes [1] that form a structure referred to as the tripartite synapse [2], [3]. When the interplay between these elements is disrupted, the regulation of synaptic signaling breaks down. Astrocyte to neuron communication is induced by the gliotransmitters glutamate, D-serine, and ATP [4]–[6]. Glutamate released by astrocytes binds to extrasynaptic neuronal NMDA receptors entraining synchronized activity [7], [8]. In the other direction, pyramidal cells signal to astrocytes primarily by glutamate that binds to astrocytic metabotropic glutamate receptors, although astrocytes are also sensitive to ATP, GABA and nitric oxide [3]. Under pathological conditions, excessive activation of astrocytes by neuronal glutamate and ATP induces paroxysmal depolarizations that initiate epileptiform discharge [9], [10]. The aim of our study was to examine whether activation of astrocyte CB1 receptors also plays a role in the generation of epileptiform discharge. A number of past observations have suggested that marijuana use may be protective against seizures [11]. The CB1 receptor exhibits the highest expression of any G protein-coupled receptor in the brain [12], and was thought to act primarily by depressing synaptic transmission [13], [14]. Recently, however, a study in the hippocampus showed that CB1 receptors also mediate the activation of astrocytes [15], which resulted in potentiated synaptic transmission [16]. Thus, CB1 signaling by astrocytes may contribute to the pathogenesis of seizures. Indeed, we find that in hippocampal networks communication from neurons to astrocytes via CB1 receptors is an essential factor in the maintenance although not the induction of epileptiform activity. Thus, endocannabinoid signaling from neurons to astrocytes represents a significant contribution to the maintenance of ED in the hippocampus.

## Methods

### Ethics statement

All animal procedures were performed in accordance with Swiss law, with strict attention given to the care and use of animals. The protocols for our experiments were approved by the Ethics Committee of the Veterinary Department of the Canton of Zurich (Approval ID 41/2011).

### Hippocampal organotypic slices

Hippocampal slice cultures were prepared from 6-day-old Wistar rats as described previously [17] following a protocol approved by the Ethics Committee of the Veterinary Department of the Canton of Zurich (Approval ID 41/2011). Briefly, hippocampi were dissected and individual transverse slices (375 µm thick) were transferred to a glass coverslip, which was placed into a test tube filled with culture medium. The tubes were then kept in a roller drum in an incubator at 36°C. After 3–4 weeks *in vitro* a coverslip bearing a hippocampal slice culture was transferred to a recording chamber on an upright microscope (Axioscope FS, Zeiss).

### Induction of epileptiform activity

4-aminopyridine (4-AP) was added to the superfusate at 100 µM, a concentration that reliably induces epileptiform activity [18]–[20]. Epileptiform activity induced in organotypic slices can be characterized according to the following three phases [18]: 1) Epileptiform discharge begins with a sharp initial depolarizing shift and then slowly returns to baseline. This initial response can be taken as time zero for data analysis. 2) During the first 30 sec of discharge, numerous high frequency/low amplitude depolarizations (tonic-like phase) are superimposed on a slow depolarizing shift. 3) During the following 10 min, epileptiform events decrease in frequency but increase in amplitude, corresponding to a clonic-like phase. These events measured intracellularly are referred to as epileptiform discharge (ED). They are quasi synchronous between CA1 pyramidal neurons and also with CA3 pyramidal neurons [21]. In extracellular recordings each epileptiform depolarization induces an “epileptic-spike”-like event in the field recording [22]–[23].

### ED quantification

**Figure 1:**
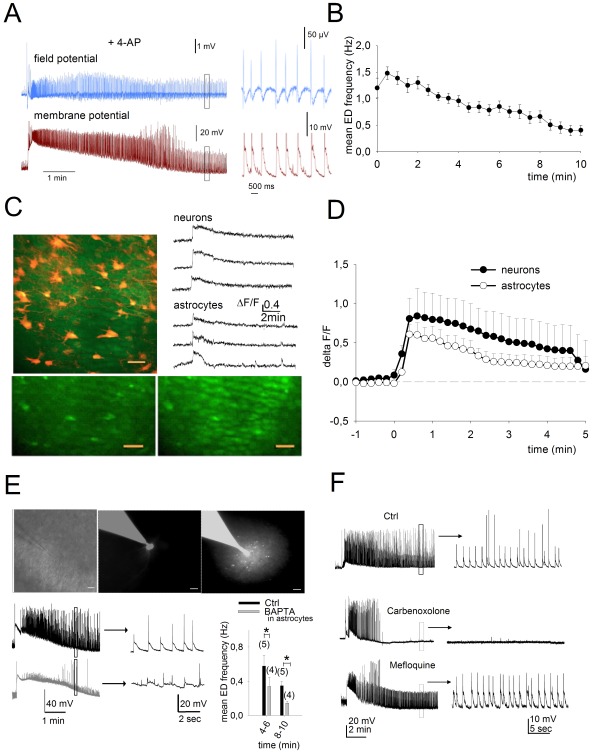
Astrocytes participate in the maintenance of epileptiform activity in CA1. A, A field recording in the CA1 area and a patch-clamp recording from a CA1 pyramidal cell show a typical initial depolarization event followed by epileptiform discharge (ED) in response to continuous application of 4-AP (100 µM). The insets show examples of individual EDs taken from the left traces at an expanded time base. B, Averaged time course of ED frequency following the initial depolarization as measured with patch-clamp recording (n = 23). C, Two-photon fluorescence imaging in hippocampal organotypic slice cultures during ED showing co-activation of neuronal and glial networks. Top right shows an overlaid image with neurons (green) loaded with OGB-1 and astrocytes (red) with sulforhodamine 101. Traces show the calcium rise both in neurons and astrocytes during 4-AP-induced epileptiform activity. Below are images of cellular calcium levels before (left) and during (right) epileptiform activity. Scale bar = 30 µm. D, Time course of fluorescence changes in CA1 pyramidal cells and in astrocytes during 4-AP superperfusion (n = 164 neurons and 146 astrocytes in 8 slice cultures). The plotted points represent averages of the mean cellular responses for each slice. E, Chelating calcium with intracellular BAPTA in astrocytes decreases the frequency of ED recorded in a CA1 pyramidal cell. Top: Images show a patch-clamp pipette approaching the CA1 stratum radiatum (left), an astrocyte patched with the pipette containing BAPTA (40 mM) and the fluorescent dye Alexa 488 (middle), and the diffusion of the fluorescent marker throughout a small network of astrocytes following depolarization (1 nA injection) to open gap-junctions (right). Bottom left: representative current clamp recordings from a CA1 pyramidal cell during ED when a neighboring astrocyte was injected with control intracellular solution (black trace) or with BAPTA (gray trace). Sample activity at expanded time base is shown at right. Bottom Right: Mean ED frequency at two time points after initiation of ED for CA1 pyramidal cells with a neighboring astrocyte filled with control intracellular solution versus a 40 mM BAPTA intracellular solution (n = 4). F, current-clamp traces recorded from single CA1 pyramidal cells in 4 different slice cultures showing ED activity in control or after incubation for three hours with two antagonists of connexins, carbenoxolone (100 µM) and mefloquine (25 µM).

To quantify the number of ED events induced by 4-AP superfusion, signals were high-pass filtered (0.05 Hz) to suppress the slow depolarizing shift, and a threshold was set above which ED events were registered. ED events were identified according to their form (polarity, amplitude, and duration). Signals were accepted as an ED if they were spontaneous (i.e. not evoked by electrical stimulation) and if they exhibited a minimum amplitude of 10 mV and minimum duration of 40 ms. We confirmed that ED events were almost synchronized between CA1 pyramidal cells (n = 5, data not shown). In addition, ED recorded in a CA1 pyramidal cell was synchronized with a simultaneous event in the field recording in CA1 stratum radiatum (n = 8, Fig. 1A). ED events were never observed in the absence of 4-AP superfusion.

### Current-clamp recordings

CA1 pyramidal neurons were recorded in the current-clamp mode of the whole cell configuration of the patch-clamp technique (Axopatch 200B amplifier, Molecular Devices). Slices were superfused continuously at a rate of 1–2 ml/min with artificial cerebrospinal fluid (ACSF) equilibrated with 95% O_2_/5% CO_2_ containing (in mM): 124 NaCl, 2.5 KCl, 26 NaHCO_3_, 1.25 NaH_2_PO_4_, 10 glucose, 3 CaCl_2_, 2 MgCl_2_, pH 7.4, at a bath temperature of 34°C. Recording pipettes (3–6 MΩ), were filled with an intracellular solution containing the following: 135 mM K-gluconate, 4 mM KCl, 10 mM HEPES, 10 mM Na_2_-phosphocreatine, 4 mM Mg-ATP, 0.3 mM Na-GTP, pH 7.2, 291–293 mOsm. Recordings were performed continuously to record the entire duration of epileptic-like activity. Electrophysiological data were acquired and analyzed with pClamp 9.0 software. Signals were analog-filtered at 10 kHz and sampled at 100–300 kHz.

### Calcium imaging

All calcium imaging experiments were performed following multi-cell bolus loading in the CA1 area of the calcium indicator Oregon Green BAPTA-1 (OGB-1 AM, 50 µg, Molecular Probes) dissolved in DMSO plus 20% Pluronic F-127 (BASF, Florham Park, NJ) to stain populations of cells. OGB-1 AM was diluted to a final concentration of 1 mM in normal rat Ringer solution containing (mM) 135 NaCl, 5.4 KCl, 5 HEPES, 1.8 CaCl_2_, at pH 7.2, and was pressure injected into the pyramidal layer of the CA1 region through a patch pipette. Two-photon calcium-imaging was performed ∼30 min after dye injection using a custom-built AOD two-photon microscope as described in [24] at 850-nm wavelength provided by a mode-locked Ti:sapphire laser system (Cameleon Ulstra II, Coherent) and a Pockels Cell (Conoptics), which allowed regulation of laser beam intensity to minimize bleaching. In all experiments, a water-immersion objective (40× LUMPlanFl/IR, 0.8 NA, Olympus) was used. Before drug application, 120 baseline images with a 2 Hz frame rate were taken over 2 minutes. Imaging was then continued at 2 Hz during 4-AP perfusion (100 µM) for 15 minutes. Imaging traces show the relative change in fluorescence (ΔF/F).

### Drugs

Concentrated stock solutions of drugs were prepared in distilled water or dimethylsulfoxide (not exceeding a final concentration of 0.02%), stored at -20°C in single-use aliquots, thawed and diluted in ACSF immediately before use. Picrotoxin, mefloquine, and carbenexolone and 4-AP were purchased from Sigma, AM251, and MPEP (2-Methyl-6-(phenylethynyl)pyridine hydrochloride) from Tocris (Bristol, UK), and tetrodotoxin (TTX), NBQX (2,3-Dioxo-6-nitro-1,2,3,4-tetrahydrobenzo[f]quinoxaline-7 –sulfonamide), CPCCOEt, WIN55212, MRS 2179 from abcamBiochemicals (Cambridge, UK), BAPTA from Molecular Probes). CGP62349 was kindly provided by Novartis AG (Basel, Switzerland).

### Statistics

Data are presented as mean percentage of control ± SEM. Unpaired Student's t-tests were used to compare responses under the various conditions. When necessary, a non-parametric statistical hypothesis test (Mann-Whitney U-test) was used to compare several samples of independent observations.

## Results

### Induction of epileptiform discharge (ED)

As approximately 70% of adult patients diagnosed with mesial temporal lobe epilepsy do not respond to drug treatment [25], we chose an *in vitro* preparation of rat hippocampal organotypic slices are exposed to 4-AP previously shown to be resistant to standard antiepileptic drugs [19]. We first confirmed that 4-AP reliably induces epileptiform discharge (ED) in rat hippocampal slice cultures under our experimental conditions. Whole cell patch clamp recordings from CA1 pyramidal cells showed that application of 4-AP (100 µM) for at least 15 min induced epileptiform discharge (ED) in 39 out of 41 slice cultures tested (Fig. 1A). As reported previously, cells responded to 4-AP with an initial tonic discharge as observed in field recordings [19], or a marked depolarization of 30.2±14.6 mV. This initial event was followed by epileptiform bursting that continued for 10.8±3.3 min. (Fig. 1A, B), also referred to as recurrent clonic-like discharges [19]. Bursts consisted of sharp depolarizations with a duration of 62.4±3.4 ms that exceeded 10 mV in amplitude (Fig. 1A) and if discharge threshold is reached, one to three action potentials were frequently elicited.

### Activity of neurons and astrocytes during ED

We examined whether astrocytes are also activated during ED by using two-photon fluorescence imaging to measure intracellular calcium signals. The membrane permeable calcium indicator Oregon Green Bapta 1-AM (OGB1-AM, 1 mM) was injected locally into slice cultures, and sulforhodamine 101 (1 µM) was applied to identify astrocytes [26]. Comparison of the OGB1-AM signal with the sulforhodamine-labeled cells in overlaid images indicated that virtually all the astrocytes in a visualized region of interest responded with an increase in calcium during ED (Fig. 1C). Calcium increases were induced in almost all neurons superfused with 4-AP (98±3%, n = 164 cells, n = 8 slices) and most astrocytes (85±15%, n = 146 cells, n = 8 slices). The amplitude of the calcium signal in neurons was delta F/F: 1±0.1 at 1–2 min and 0.63±0.12 at 5 min and in astrocytes it was delta F/F: 0.4±0.04 at 2 min and 0.12±0.03 at 5 min (Fig. 1D). The duration of elevated calcium signals in neurons corresponded to the time course of ED measured in patch-clamp recordings from CA1 pyramidal cells (Fig. 1B, D).

### Role of astrocytes in ED initiation

To determine whether the calcium elevations in astrocytes represent a correlative response or a causal determinant of ED, the effect of inhibiting calcium signaling in astrocyte networks was evaluated. Astrocytes were patched in the stratum radiatum of CA1 close to the stratum pyramidale. Calcium was chelated in a targeted astrocyte patched with a pipette containing BAPTA (40 mM) as well as the fluorescent dye Alexa 488, which allowed us to monitor diffusion of the pipette contents. Cells were considered to be astrocytes based on their negative resting potential (−90±4 mV), their low membrane resistance (18±5 MΩ, n = 8), and the absence of action potentials in response to current injection. By depolarizing the patched astrocyte, gap junctions are opened, resulting in the diffusion of the pipette contents to neighboring astrocytes in the syncytium [27]. When a recording was now obtained from a neighboring CA1 pyramidal cell, the frequency of events during ED was significantly lower than that measured in a CA1 pyramidal cell in which an adjacent astrocyte was patched with a pipette containing the standard recording solution without BAPTA (ED decreased by 58±6%, n = 4, *p*<0.05, after 8–10 min, Fig. 1E). Similarly, blocking gap junctions with carbenoxolone (100 µM), reduced the duration of epileptiform activity, as reported previously [27], but not with mefloquine (Fig. 1F).

**Figure 2:**
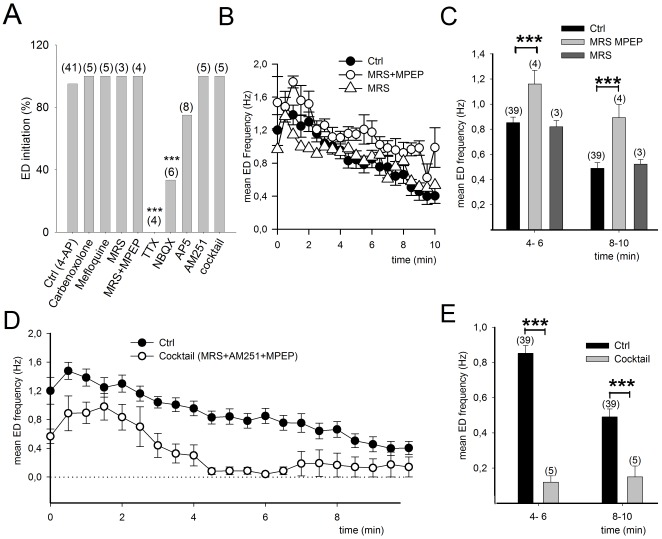
The duration but not the initiation of ED is reduced following disruption of neuroglial interactions. A, Compounds reported to disrupt neuroglial and astrocyte coupling do not modify the initiation of ED in response to 4-AP. The probability of ED initiation was not changed either by antagonists for gap junctions or by a solution that inhibits neuroglial interactions consisting of MRS 2179 (4 µM) to block P2Y1 receptors and MPEP (10 µM) to block mGluR5. The probability of ED initiation was not changed either by a cocktail containing antagonists for P2Y1R, mGluR5 and CB1R (AM251, 4 µM) or CB1R antagonist alone. But ED initiation is prevented when synaptic transmission is blocked with TTX (1 µM), or reduced when ionotropic glutamate receptors are blocked with NBQX (50 µM). B, Application of antagonists for P2Y1 and mGlu5 receptors increases the frequency of ED induced by 4-AP, while MRS alone has not effect. C, Mean ED frequency 4–6 and 8–10 minutes after initiation. D, E, Addition of AM251 to the MRS 2179 and MPEP solution significantly reduces ED frequency.

Although these experiments show that astrocytes significantly contribute to ED, their activity did not appear to modulate the initiation of the 4-AP-induced discharge. To address this issue in more detail, the effects of several known inhibitors of glial function on the probability of ED initiation in response to 4-AP was quantified. Carbenoxolone (100 µM), a blocker of neuronal as well as glial gap junctions, did not alter the probability of initiation of ED (0 out of 5 slices), nor did mefloquine (25 µM, 0 out of 5 slices), a blocker primarily of neuronal gap junctions composed of Cx36 and Cx50 [28], [29] (Fig. 2A). A combination of antagonists reported to inhibit neuroglial interactions by blocking P2Y1 (MRS 2179, 4 µM), and mGlu5 (MPEP 10 µM) receptors [8], [30], [31], [32] also failed to modify initiation of ED (0 out of 4 slices, Fig. 2A). Initiation of ED was, however, prevented when synaptic transmission was inhibited with TTX (1 µM) (4 out of 4 slices, p<0.001, ANOVA), significantly reduced by NBQX (50 µM) (4 of 6 slices, p<0.001) but less affected by the NMDAR antagonist AP5 (40 µM) (2 out of 8 slices, p>0.05; Fig. 2A).

### Role of astrocytes in ED maintenance

As the initiation of ED appears to be independent of astrocyte activation, we then tested whether the maintenance of recurrent discharge is affected by inhibitors of neuroglial communication. Neurons can activate astrocytes by releasing glutamate ([33], [34] that acts mainly on mGluR5 [8] and ATP that acts on P2Y1 receptors ([30]–[32]. However, neither the selective blockade of P2Y1 receptors with MRS 2179 (4 µM) (6±15%, n = 3, *p*>0.1), nor the combined blockade of P2Y1 and mGlu5 receptors (MRS 2179, 4 µM+MPEP, 10 µM; 30±6%, n = 4, *p*<0.001) reduced the frequency of ED events (Fig. 2B, C). In fact, the addition of MPEP to the superfusate led to an increase in ED frequency, as was also reported previously [10] during interictal activity induced by picrotoxin/zero-magnesium in acute slices of entorhinal cortex.

### Role of interneuronal and astrocytic CB1R in ED maintenance

**Figure 3:**
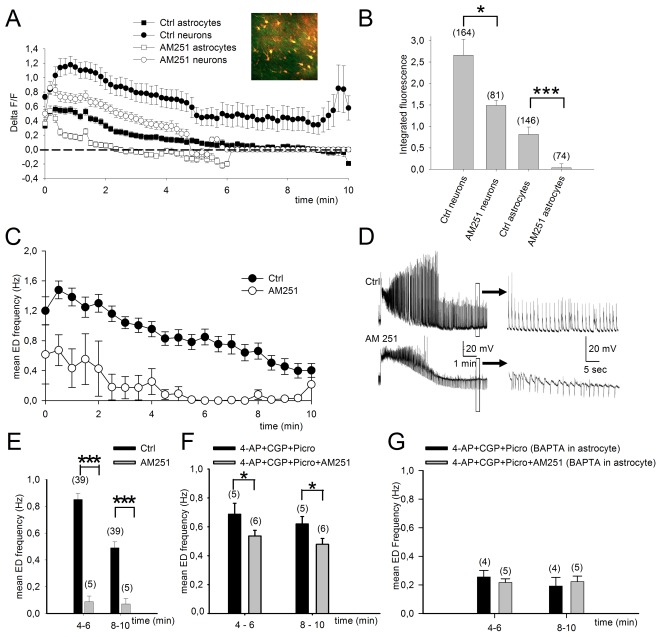
CB1 receptors expressed by astrocytes contribute significantly to maintenance of ED in CA1. A, Two-photon fluorescence calcium imaging of CA1 cells during 4-AP perfusion of the slice. Fluorescence signals from neurons and astrocytes during 4-AP in the presence or absence of the CB1 receptor antagonist AM251 (4 µM). The plotted points represent the averages of the responses for each individual cell. B, Blocking CB1 receptors significantly reduced integrated area of calcium induced fluorescence measured 1–4 min after ED initiation. This inhibition was markedly particularly important in astrocytes (p<0.001 Mann-Whitney U test) C, Similarly, average time course show that ED in CA1 pyramidal cells recorded in current-clamp mode is reduced in the presence of the CB1 antagonist AM251. D, Representative traces for data shown in C. E, Mean ED frequency at two time points after initiation of ED for CA1 pyramidal cells show that the reduction induced by AM251 is highly significant. F, In the presence of GABA_A_ and GABA_B_ receptor antagonists to block interneuron signaling, blocking CB1 receptors still results in a significant reduction in ED but much less effective in reducing ED frequency. G, When astrocyte signaling is inhibited by BAPTA injection (40 mM) under conditions where GABAergic transmission is blocked, the blockade of CB1 receptors no longer reduces 4-AP-induced ED.

Recent findings demonstrate that astrocyte signaling is also sensitive to endocannabinoids released from neighboring neurons [15], [16]. We therefore tested the effect of adding AM 251 (4 µM), a CB1 receptor antagonist, to the superfusate containing the antagonists for CB1, P2Y1 and mGlu5 receptors. Neither this cocktail nor the CB1 antagonist alone, inhibited epileptiform discharges initiation (0 out of 5 slices, Fig. 2A). But instead of increasing ED, this solution significantly reduced their frequency (−85±5%, n = 5, *p*<0.001; Fig. 2D, E). As these results indicate that activation of hippocampal CB1 receptors represents a prominent determinant in the entrainment of recurrent ED induced by 4-AP, we focused the remainder of our experiments on characterizing the mechanisms involved in endocannabinoid signaling during ED. Selective blockade of CB1 receptors with AM 251, diminished calcium signals in CA1 pyramidal neurons (by 43±4%, n = 81, *p*<0.05, 1–4 min, Fig. 3A, B). This inhibition was markedly greater in astrocytes than in neurons with a decrease in the frequency of ED events by 95±11% (n = 74, p<0.001 Mann-Whitney U-test). This difference in response between neurons and astrocytes suggest that activation of astrocytes via CB1 receptors is particularly pronounced during epileptiform activity. A comparable effect on 4-AP-induced ED in response to blockade of CB1 receptors was observed in current clamp recordings in CA1 pyramidal cells (−89±5, n = 5, *p*<0.001, Fig. 3C, D, E). Interestingly, blockade of CB1 receptors not only attenuated ED, but also revealed the presence of repetitive, large amplitude inhibitory post-synaptic potentials. This observation suggests that the intense activity generated by 4-AP leads to depolarization-induced suppression of inhibition (DSI), as described previously [35], and that the inhibition of DSI by blocking CB1 receptors [36] allows GABAergic synaptic signaling to reemerge.

As CB1 receptors in the hippocampus are expressed not only by astrocytes but also by the CCK-positive subpopulation of GABAergic interneurons [14], we re-examined the effect of CB1 receptor blockade on ED frequency under conditions where interneuron signaling was prevented by inhibiting GABA_A_ (picrotoxin) and GABA_B_ receptors (CGP62349). Under these conditions, blockade of CB1 receptors with AM 251 still led to a significant reduction in ED (4-AP+CGP+Picro+AM251, n = 6, compared to 4-AP+CGP+Picro, n = 5; decrease of −23±5% *p*<0.05) although this effect was much less than with interneuron signaling intact (−89±5%; Fig. 3F). Thus, both astrocytes and interneurons are involved in the CB1 receptor-mediated maintenance of ED. To further confirm a contribution from astrocytes to the CB1 receptor-dependent enhancement of ED maintenance, we quantified the effects of CB1 receptor blockade after both interneuron and astrocyte signaling were inhibited with GABA antagonists and intracellular BAPTA, respectively. As expected, application of AM251 now no longer affected ED (−16±17%, n = 5, p>0.5; Fig. 3G).

## Discussion

The concept of the tripartite synapse in which astrocytes exert an essential function in controlling synaptic transmission is now well established [5]. In the case of excessive neuronal discharge generated by epileptiform bursts a commensurate powerful activation of astroglial networks is induced [27]. A recent study has shown that astrocytes respond primarily to glutamate and ATP during neuronal hyperactivity, which induces them, in turn, to release glutamate onto neurons resulting in a positive feedback loop that amplifies the initiating neuronal event [10]. Our results in an in vitro model of drug resistant epilepsy show that endocannabinoid signaling from neurons to astrocytes represents a further significant mechanism that promotes the maintenance of epileptiform activity in the CA1 hippocampal network. We found that blocking CB1 receptors during 4-AP-induced epileptiform activity markedly diminished calcium responses in astrocytes and the frequency of ED recorded in CA1 pyramidal cells. However, CB1 receptor blockade did not reduce the probability of initiation of epileptiform activity. Our findings are consistent with the expression of CB1 receptors in hippocampal astrocytes, and the demonstration that these receptors are targeted by endocannabinoids released from pyramidal cells [15]. Activation of astrocytic CB1 receptors then induces release of calcium from intracellular stores triggering glutamate release, which potentiates synaptic transmission in neighboring neurons [15], [16]. However in the present study we did not discriminate between glutamate released by astrocytes and glutamate released by neurons during the epileptic-like activity. To date, there are no pharmacological tools that specifically inhibit glutamate release from astrocytes without affecting glutamate release from neurons.

A more prominent action of endocannaboids in the hippocampus is DSI of cholecystokinin-positive interneurons [14], leading to reduced release of GABA [37], and also depression at excitatory glutamatergic terminals but, in the hippocampus, to a much lesser extent [38]. In our *in vitro* model the disinhibition resulting from endocannabinoid-mediated DSI of interneurons represents a major contribution to the maintenance of epileptiform activity (Fig. 3E versus Fig. 3F). Nevertheless, when GABAergic signaling by interneurons was blocked, a significant endocannabinoid-dependent response mediated by astrocytes remained (Fig. 3G). Thus, endocannabinoid signaling from pyramidal cells to both astrocytes and CB1 receptor-positive interneurons is involved in the maintenance of epileptiform activity in our experiments. In this respect, it is interesting to note that in control conditions, a very strong temporal and spatial correlation was observed between endocannabinoid-mediated DSI and astrocytic calcium elevation in response to neuronal discharge [15].

Additional mechanisms through which astrocytes may contribute to epileptiform activity include the modulation of neurovascular coupling and of glucose trafficking in response to the intense activity during seizures [10], [27]. Furthermore, the intense neuronal activity generated by epileptiform bursts increases the functional extent astrocytic networks, an effect mediated by gap junctions [27]. We also found evidence for an involvement of gap junction signaling among astrocytes in the maintenance of epileptiform activity.

Our main finding, that endocannabinoids promote the maintenance of epileptiform activity may seem surprising in light of several earlier investigations. In the *in vitro* kainate model of epileptogenesis, it appears that contrary to control conditions [38] endocannabinoids induce proportionally greater inhibition of glutamate release from pyramidal cells than GABAergic release from interneurons, thus having a net suppressing effect on seizure-like activity [39]; [40]. Similarly, in a low magnesium slice model of hippocampal seizures, activation of CB1 receptors decreased the frequency of ED [41]. In *in vivo* studies, stimulation of CB1 receptors had anticonvulsant effects in the electroshock model to evoke seizures in mice [42] and in the rat pilocarpine model of epilepsy [43]. And in rat neonates, blocking CB1 receptors resulted in epileptic activity [44]. In contrast, chronic blockade of CB1 receptors was found to reduce the seizure threshold induce by hyperthermia [45]. Also, studies in humans have reported both pro and anticonvulsive effects of smoking marijuana in subject with epilepsy [11], whereas other work suggests that activation of CB1 receptors does not alter the propensity for seizures [46]. The discrepancies between these results obtained in animal and human studies suggest that the modulation CB1 receptors has complex actions, and perhaps opposite effects on neuronal versus glial network activity. Activation of CB1 receptors may be useful in counteracting the initiation of seizures by promoting depolarization-induced suppression of excitation. However, as shown by our data, activation of CB1 receptors expressed by astrocytes triggers calcium signaling in glial networks that in turn can amplify responses of excitatory neurons and thus enhance the maintenance of epileptiform discharge.
